# Validation of a 3D‐printed robot‐assisted partial nephrectomy training model

**DOI:** 10.1002/bco2.269

**Published:** 2023-08-28

**Authors:** Thomas Hermans, Joren M. Snoeks, Frank vom Dorp, Christoph Wiesner, Thomas Steiner, Friedrich‐Carl von Rundstedt

**Affiliations:** ^1^ Department of Urology Helios University Hospital Wuppertal, University of Witten/Herdecke Wuppertal Germany; ^2^ Community Ecology Lab, Department of Biology Vrije Universiteit Brussel (VUB) Brussels Belgium; ^3^ Helios Klinikum Duisburg Duisburg Germany; ^4^ Helios Klinikum Salzgitter Salzgitter Germany; ^5^ Helios Klinikum Erfurt Erfurt Germany

**Keywords:** 3D‐printed, GEARS score, partial nephrectomy, robot‐assisted surgery, training model, validity

## Abstract

**Objectives:**

Most renal tumours can be treated with a partial nephrectomy, with robot‐assisted partial nephrectomy becoming the new gold standard. This procedure is challenging to learn in a live setting, especially the enucleation and renorraphy phases. In this study, we attempted to evaluate face, content, and preliminary construct validity of a 3D‐printed silicone renal tumour model in robotic training for robot‐assisted partial nephrectomy.

**Materials and Methods:**

We compared the operative results of three groups of surgeons with different experience levels (>20 partial nephrectomies, 1–20 partial nephrectomies and no experience at all) performing a robotic tumour excision of a newly developed silicone model with four embedded 3D‐printed renal tumours. We evaluated the participants' performance using surgical margins, excision time, total preserved parenchyma, tumour injury and GEARS score (as assessed by two blinded experts) for construct validity. Postoperatively, the participants were asked to complete a survey to evaluate the usefulness, realism and difficulty of the model as a training and/or evaluation model. NASA‐TLX scores were used to evaluate the operative workload.

**Results:**

Thirty‐six participants were recruited, each group consisting of 10–14 participants. The operative performance was significantly better in the expert group as compared to the beginner group. NASA‐TLX scores proved the model to be of an acceptable difficulty level.

Expert group survey results showed an average score of 6.3/10 on realism of the model, 8.2/10 on the usefulness as training model and 6.9/10 score on the usefulness as an evaluation tool. GEARS scores showed a non‐significant tendency to improve between trials, emphasizing its potential as a training model.

**Conclusion:**

Face and content validity of our 3D renal tumour model were demonstrated. The vast majority of participants found the model realistic and useful for training and for evaluation. To evaluate construct and predictive validity, we require further research, aiming to compare the results of 3D‐model trained surgeons with those of untrained surgeons in real‐life surgery.

## 1 INTRODUCTION

Renal cancer is a frequent pathology, composing about 2%–3% of all malignant diseases and responsible for circa 180 000 deaths per year.^1^ In localized T1 (and small T2) renal cancers, the recommended treatment is partial nephrectomy, since it is associated with a higher overall survival rate and better postoperative kidney function than radical nephrectomy.^2^, ^3^ Over the past decade, technological improvement caused a shift towards minimally invasive and robot‐assisted techniques for partial nephrectomy (RAPN).

Usually during this procedure, the renal artery is clamped while the tumour is resected to limit blood loss, challenging surgeons to keep warm ischemia times as short as possible in order to preserve renal function and improve overall survival. Except for time pressure when clamping, this procedure is also difficult to learn because of the variable vascular access, aim to preserve as much renal parenchyma as possible, risk of severe bleeding during resection and the necessity of avoiding tumour injury or spillage. The steep learning curve for this technique and the challenges of actually getting hands‐on experience have created the need for an effective and realistic surgical training model.^4^, ^5^

Existing training modalities can be summarized in three groups: biological models (animal tissue, live animals or human cadavers), non‐biological (bench‐top) models and digital (virtual reality) simulators. Several techniques for creating in and ex vivo renal pseudotumours in porcine kidneys already do exist,^6^, ^7^, ^8^ all of them posing their own problems such as adhesion problems, extravasation of the injected pseudotumour material, poor tumour margins or being isoechoic on ultrasound.

3D silicone tumour models have the advantage over biological tissue of being more cost‐effective and having a nearly unlimited shelf‐life, which clearly facilitates logistics. They are available and ready when needed.

Several of these pilot models have been rated highly realistic, but face, construct and content validity have not been effectively studied.^9^, ^10^

**FIGURE 1:**
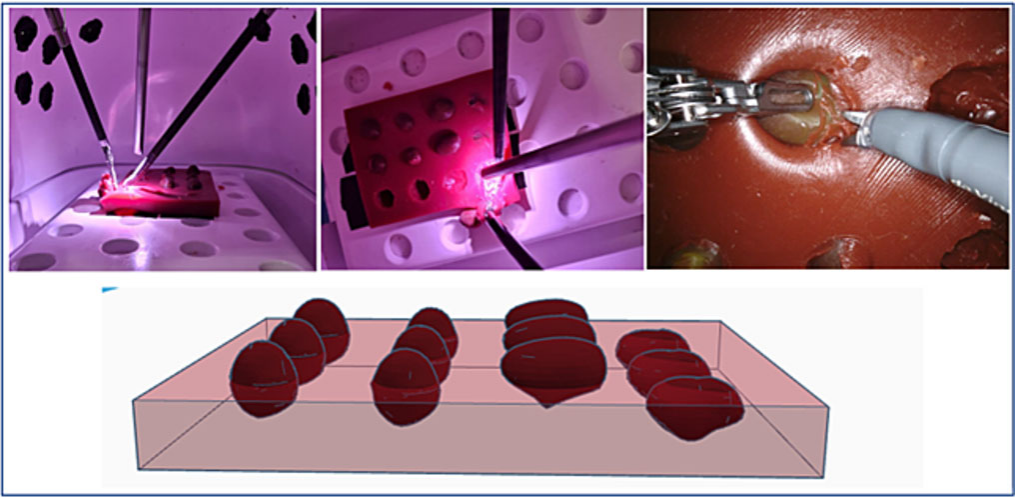
In‐training images of a robotic enucleation and a 3D sketch of the model.

In this study, we evaluate a new model consisting of four different 3D‐printed silicone tumour models in a silicone sheet, created by Lazarus 3D, LLC (Houston, TX, USA) and based on 3D‐analysis of real‐life renal tumours. We have aimed to demonstrate face, content and construct validity of the model in order to promote 3D silicone tumour models in robotic training for RAPN and especially for tumour enucleation (Figure 1).

## 2 MATERIALS AND METHODS

### 2.1 3D Model

The model used was a silicone sheet consisting of three rows of four different 3D‐printed silicone tumour models, thus containing 12 tumours in total. They were printed in a soft biomimicking synthetic tissue, with a slightly harder silicone for the tumour capsula and interior.

The tumours were 1:1 copies of MRI images of four different real‐life renal tumours. These were selected as they represented typical cases from our clinical experience. The tumours were designed with a 1 mm capsule in a brown colour surrounding yellow tumour tissue and were imbedded in a sheet of dark red silicone. The first two tumours were comparable and both around 50% endo/exophytic. The third was largely exophytic and the last tumour was largely endophytic. All were 1.5–3 cm in diameter. Partial R.E.N.A.L. nephrometry scores for each tumour were thus 5, 5, 3 and 7 out of 9, respectively. Location relative to the polar lines and anterior/posterior location relative to the coronal plane at the hilar vessels could not be determined, since the design of the model would not permit this (tumours imbedded in a rectangular silicone sheet and not in an actual kidney).

The models were created by Lazarus 3D Inc. (Albany, OR, USA).

### 2.2 Participants

Participants were recruited between December 2021 and June 2022 among urology residents and attending urologists with or without experience in robotic surgery in nine different German centres. These were divided into three a priori defined participant groups of different experience levels: no experience in RAPN, 1–20 RAPN as a primary surgeon and >20 RAPN as a primary surgeon.

### 2.3 Simulation

The participants were asked to fill out a demographic survey, then perform a robotic resection of four consecutive kidney tumours of increasing difficulty. Afterwards, all participants were asked to provide answers to a standardized evaluation of the model.

All participants received the same oral instructions before starting the simulation. Surgical systems used were the Da Vinci Si, X and Xi (Intuitive Surgery) with training instruments and a 30° camera, using only three arms. Surgeons were allowed to choose between a ProGrasp forceps or a Maryland bipolar forceps. The simulations were recorded on video.

### 2.4 Outcome parameters

Demographic data were collected using a questionnaire (age, sex, training level, experience levels).

Face and content validity were assessed using several survey questions about difficulty, comparability to the reality and usefulness of the models with ordinal 10‐point rating Likert scales (where 1 was strongly no and 10 was definitely yes). We assessed perceived operative workload with the Nasa Task Load Index (NASA‐TLX),^11^ a model developed in the late 1980s to evaluate task demands (subjective physical demands, mental demands, temporal demands, frustration, effort and performance) by Hart et al. and validated numerous times since. We defined an acceptable workload as a NASA‐TLX score of less than 56.5, which was the average score for intense video gaming in Grier's 2015 meta‐analysis, other reference points being 46 for executing cognitive tasks and 62 for physical activities.^12^

Participants had the opportunity to comment on their experience.

Surgical parameters were assessed separately per tumour based on inspection and weight of the resected tissue and based on the video analysis: resection time, R‐status, depth of tumour incision, amount of excess tissue resected, spillage of tumour content (if the tumour tissue was incised, i.e., yellow was visible), injury to the tumour capsule (if the capsule was incised, i.e., brown was visible) and entry of the collecting system (perforation of the silicone sheet).

See Table A1 for the units of measurement for each investigated parameter.

In order to calculate the depth of tumour incision, the closest point to the tumour capsula (negative value) or the deepest incision in the tumour was measured (positive value) using a millimetric point‐shaped ruler. The amount of excess tissue resected was calculated with a precision weighing scale with a resolution of 0.01 g.

Expert‐assessed surgical performance was measured for the first and last tumour in a random order using the standardized and validated Global Evaluative Assessment of Robotic Surgeons (GEARS).^13^ This was done independently by two experienced investigators, who were blinded to tumour number and participant. Videos were anonymized and contained no audio.

### 2.5 Statistics

Statistical analyses were done by a blinded independent investigator. Data analysis was carried out in R v4.2.1 (R Core Team, 2020) using specified packages and functions (hereafter *package:function*). In order to assess face, content and construct validity, and operative demand, we compared the outcome parameters (Table 1) between groups of participants from different experience levels with linear models (*stats:lm*). For outcome parameters that were measured over trials, linear mixed models were constructed with trial and individual as random factors (*lme4:lmer*). When the assumption of normality was violated (*stats:shapiro.test*), generalized linear mixed models (*lme4:lmer*), or non‐parametric equivalents (*stats:kruskal.test*) were used. For linear (mixed) models and Kruskal–Wallis tests, homoscedasticity was assessed (*car:ncvTest* and *onewaytests:bf.test*, respectively). For generalized linear mixed models, overdispersion was assessed by modelling with and without observation‐level random effects and subsequently comparing both models by Akaike's Information Criterion (*stats:AIC*). Tukey's post hoc test (*multcomp:glht*) or Dunn's test (*FSA:dunnTest*), where parametric assumptions were violated, was used to test for pairwise differences between groups.

**TABLE 1:** Participant demographic and experience information by participant groups. Simulation experience was measured in hours of robotic simulation (digital or ex vivo). Bedside assisting experience was measured as the number of RAPN as assistant surgeon. Robot experience was measured as the general number of robot‐assisted operations as primary surgeon. RAPN experience was measured as the number of RAPN as primary surgeon.

Experience level	No experience	Beginner	Expert
*N*	14	12	10
Age (mean ± SE)	35.7 ± 2.5	37.8 ± 1.9	46.3 ± 1.8
Male	12	8	10
Female	2	4	0
Students	1	0	0
Residents	9	1	0
Urologists	4	11	10
Simulation experience (mean ± SE)	11.0 ± 7.3*	19.8 ± 8.0^+^	/
Bedside assisting experience (mean ± SE)	91.4 ± 36.2	116.0 ± 37.7	222.8 ± 108.7^†^
Robot experience (mean ± SE)	10.7 ± 10.3	25.8 ± 5.9	1025.5 ± 319.5
RAPN experience (mean ± SE)	0 ± 0	5.4 ± 1.1	215.0 ± 70.1

Abbreviation: RAPN, robot‐assisted partial nephrectomy.

* *n* = 13;

^+^ *n* = 5;

^†^ *n* = 8.

### 2.6 Ethics

All data were collected anonymously. Participants were referred to using ID numbers.

The study was approved by the ethical committee of the University of Witten‐Herdecke.

## 3 RESULTS

### 3.1 Participants

We recruited 36 participants from nine different hospitals, among which were one surgical assistant, 10 urology residents and 25 board certified urologists. After being categorized based on the level of experience, each group included at least 10 participants: no experience (*n* = 14), beginner (*n* = 12) and expert (*n* = 10).

Mean number of RAPN as a primary surgeon was 5.4 ± 1.1 in the intermediate group and 215.0 ± 70.1 in the expert group. Participant demographics and experience levels are shown in Table 1.

### 3.2 Face validity (comparability)

**FIGURE 2:**
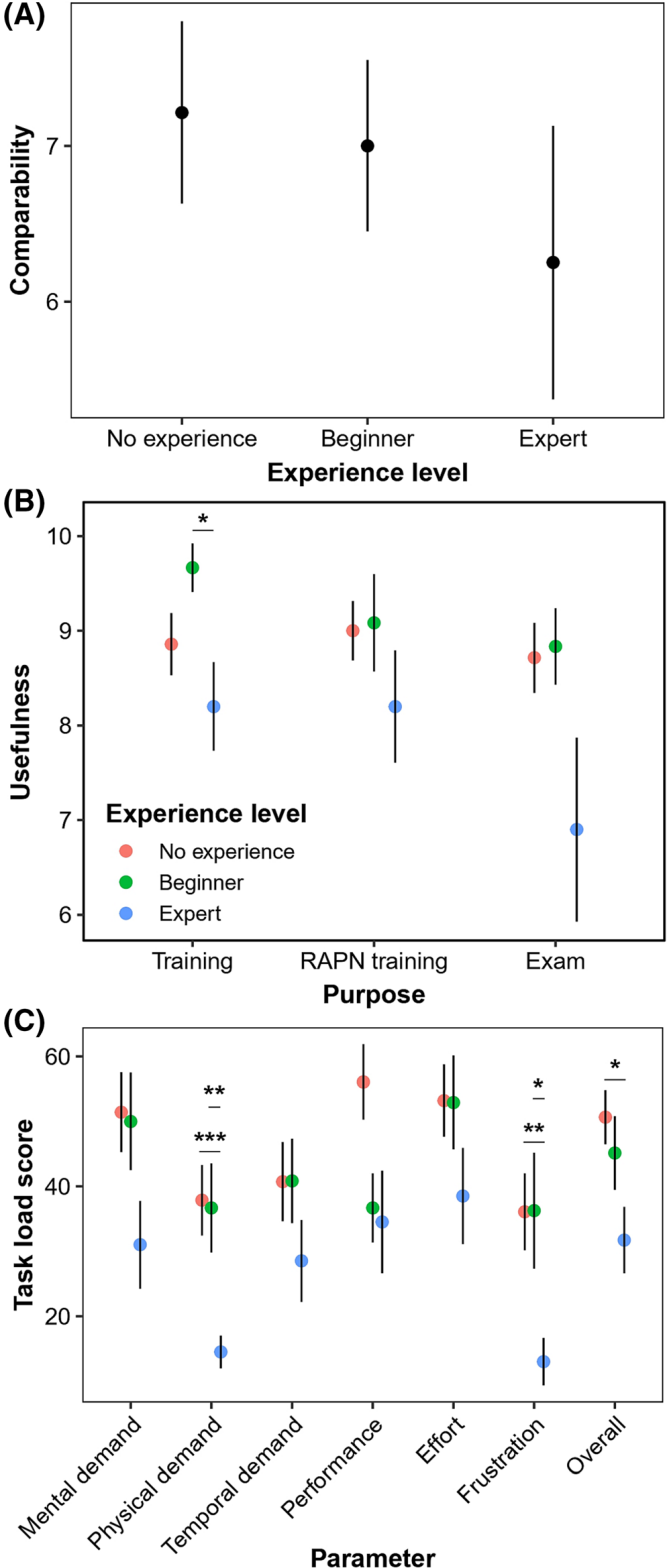
Mean ± standard error results, as compared between participant groups, of the (A) comparability of the model to reality, (B) the usefulness of the model for robotic training in general, for robot‐assisted partial nephrectomy (RAPN) training specifically, and as an examination tool, and (C) Nasa Task Load Index (NASA‐TLX) scores for each task load subcategory. Scores were given on a scale of 0 to 10. *: significant differences between participant groups with *p* < 0.001.

The overall comparability of the model to reality was rated 6.9 ± 0.4 out of 10 (Table 2), with no significant differences between the participant categories (Figure 2A and Table A2).

**TABLE 2:** Mean ± standard error results of the operative demand and the content, face, and construct validity of the 3D‐printed RAPN training model by participant group. Operative demand was scored according to the NASA‐TLX model (Hart et al.^11^). Content validity and face validity was scored by each participant on a scale to 10. GEARS scores were blindly assessed by two separate experts, with scores ranging from 1 to 25. Percentages indicate proportion of participants that left tumour tissue in situ (R1), that caused tumour spillage, that caused tumour capsula injury, or that perforated the virtual renal pelvis (bottom of the model), respectively.

Experience level	No experience	Beginner	Expert	Overall
OPERATIVE DEMAND				
Mental demand	51.4 ± 6.1	50.0 ± 7.5	31.0 ± 6.7	45.3 ± 4.1
Physical demand	37.9 ± 5.4	36.7 ± 6.9	14.5 ± 2.5	31.0 ± 3.6
Temporal demand	40.7 ± 6.1	40.8 ± 6.5	28.5 ± 6.3	37.4 ± 7.5
Performance	56.1 ± 5.8	36.7 ± 5.3	34.5 ± 7.9	43.6 ± 7.9
Effort	53.2 ± 5.6	52.9 ± 7.2	38.5 ± 7.4	49.0 ± 7.9
Frustration	36.1 ± 5.9	36.3 ± 8.9	13.0 ± 3.7	29.7 ± 8.5
Overall	50.7 ± 4.2	45.1 ± 5.7	31.7 ± 5.1	43.6 ± 6.3
CONTENT VALIDITY				
Training usefulness	8.9 ± 0.3	9.7 ± 0.3	8.2 ± 0.5	8.9 ± 0.2
RAPN training usefulness	9.0 ± 0.3	9.1 ± 0.5	8.2 ± 0.6	8.8 ± 0.3
Exam usefulness	8.7 ± 0.4	8.8 ± 0.4	6.9 ± 1.0	8.3 ± 0.4
FACE VALIDITY				
Comparability	7.2 ± 0.6	7.0 ± 0.6	6.3 ± 0.9	6.9 ± 0.4
CONSTRUCT VALIDITY				
GEARS	13.8 ± 0.8	17.8 ± 0.6	22.6 ± 0.4	17.7 ± 0.5
CONTENT VALIDITY				
Depth of tumour incision (mm)	−0.6 ± 0.5	0.4 ± 0.5	0.7 ± 0.6	0.1 ± 0.2
Total excised parenchyma (g)	3.2 ± 0.1	3.0 ± 0.1	3.0 ± 0.1	3.1 ± 0.1
Resection time (min)	5.2 ± 0.6	6.2 ± 0.6	3.3 ± 0.4	5.0 ± 0.3
Residual tumour tissue (%)	26.8 ± 0.8	6.3 ± 0.5	0.0 ± 0.0	12.5 ± 0.2
Tumour spillage (%)	55.4 ± 0.9	31.3 ± 1.0	17.5 ± 0.9	36.8 ± 0.3
Tumour capsula injury (%)	64.3 ± 0.9	52.1 ± 1.0	25.0 ± 1.0	49.3 ± 0.3
Renal pelvis perforation (%)	19.6 ± 0.7	22.9 ± 0.9	12.5 ± 0.8	18.8 ± 0.3

Abbreviations: GEARS, Global Evaluative Assessment of Robotic Surgeons; NASA‐TLX, Nasa Task Load Index; RAPN, robot‐assisted partial nephrectomy.

### 3.3 Difficulty

**FIGURE A1:**
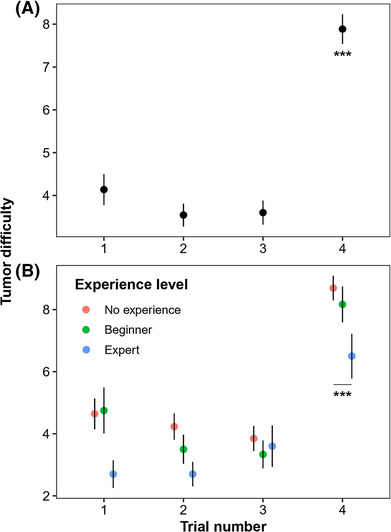
Mean ± standard error of the difficulty of each tumour, as scored by each participant on a scale of 0 to 10, by participant group. ***: significant difference in the rated tumor difficulty between participant groups (*p* < 0.001).

Average difficulty of the first three tumour models was similar, with scores between 3.5 and 4.1/10 (Table A3). Tumour 4, which was more endophytic, was rated to be significantly more difficult, with a mean difficulty of 7.9 ± 0.3 (Figure A1A and Table A4). In addition, Tumour 4 was rated significantly easier by experts than by the other two groups (*p* = 0.03; Figure A1B).

Perceived workload of the task was assessed through NASA‐TLX scores. Mean overall scores were 50.7 ± 4.2, 45.1 ± 5.7 and 31.7 ± 5.1 for each group, respectively (Table 2). These scores were all below the pre‐defined acceptable workload score of 56.5, thus rendering the workload of using the model acceptable for users. Experts scored the trials significantly lower on overall operative demand than beginners and significantly lower on physical demand and frustration than both other participant categories (Figure 2C).

### 3.4 Content validity (usefulness and quality)

Participants rated the model very useful, with scores for robotic training in general, RAPN training or as an examination tool being 8.9 ± 0.2, 8.8 ± 0.3 and 8.3 ± 0.4, respectively (Tables 2 and A4). Experts rated the model less useful for robotic training in general than both other groups (*p* = 0.01; Figure 2B). For partial nephrectomy training and as an examination tool, there were no statistical differences between the groups (Table A2).

**FIGURE 3:**
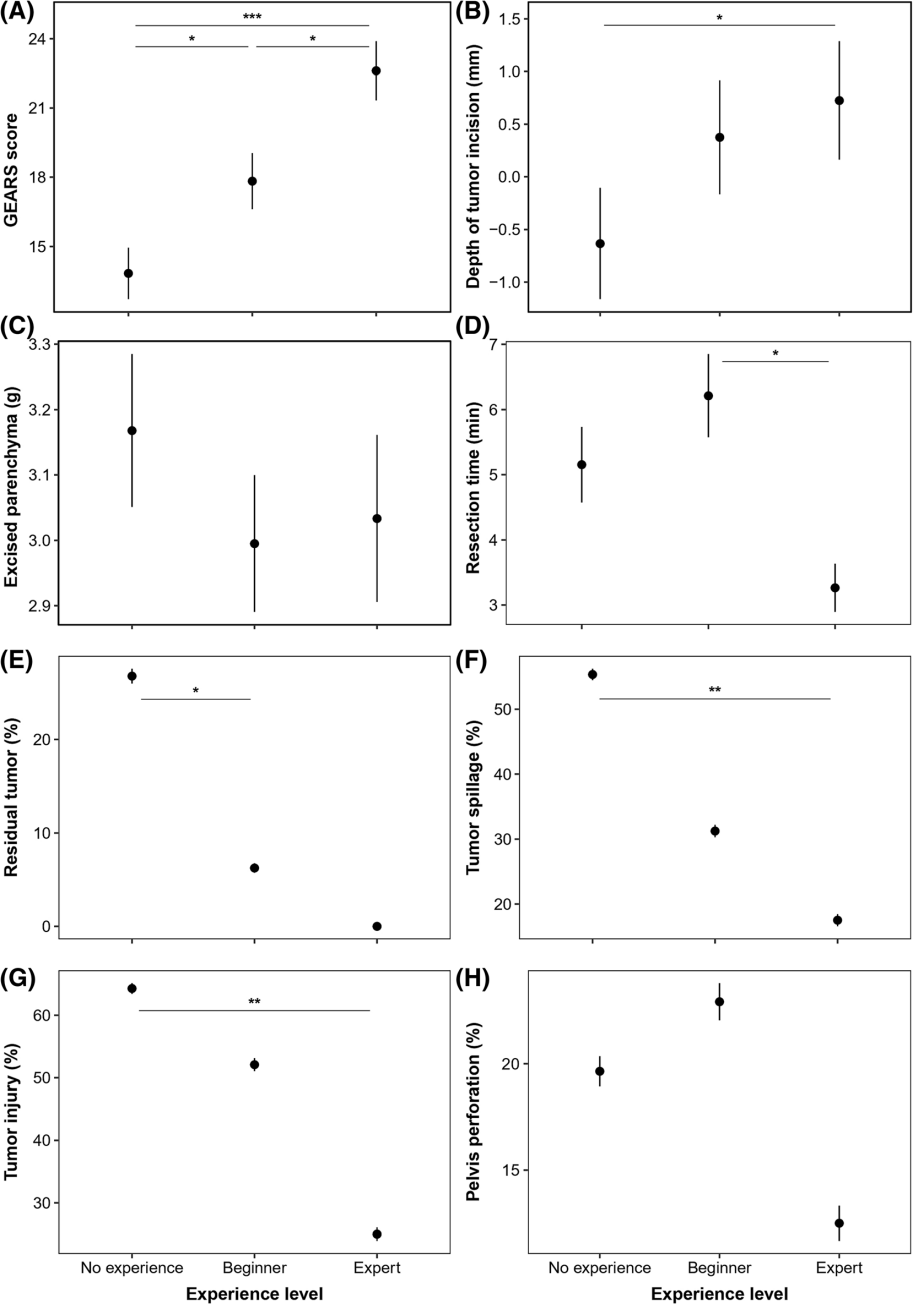
Overview of the mean ± standard error results of the operative outcomes as compared between participant categories. *: significant differences between the operative outcome of different participant groups with p.

We did an extensive statistical analysis of the surgical outcomes, comparing the three participant groups with each other. As expected, the expert group had significantly less residual tumour tissue (=R1, *p* < 0.001), less superfluously excised ‘kidney’ parenchyma (*p* < 0.001) and shorter resection times (*p* < 0.001). The expert group also caused significantly less injury to the capsule (*p* < 0.01) or tumour spillage (*p* < 0.01) and perforated the silicone sheet les frequently (however, not statistically significant, *p* = 0.38). Additionally, none of the experts left any residual tumour tissue. All results are summarized in Figure 3. For more detailed results, see Tables 2 and A2.

The expert group also had GEARS scores that were associated with the level of experience resulting in higher scores for experts in comparison to beginners, and higher scores for beginners in comparison to participants without any prior experience (Table A2 and Figure 3). There was no statistical difference (F = 2.00; *p* = 0.17) between the reported GEARS scores of both experts.

### 3.5 Construct validity (predictive value)

**FIGURE 4:**
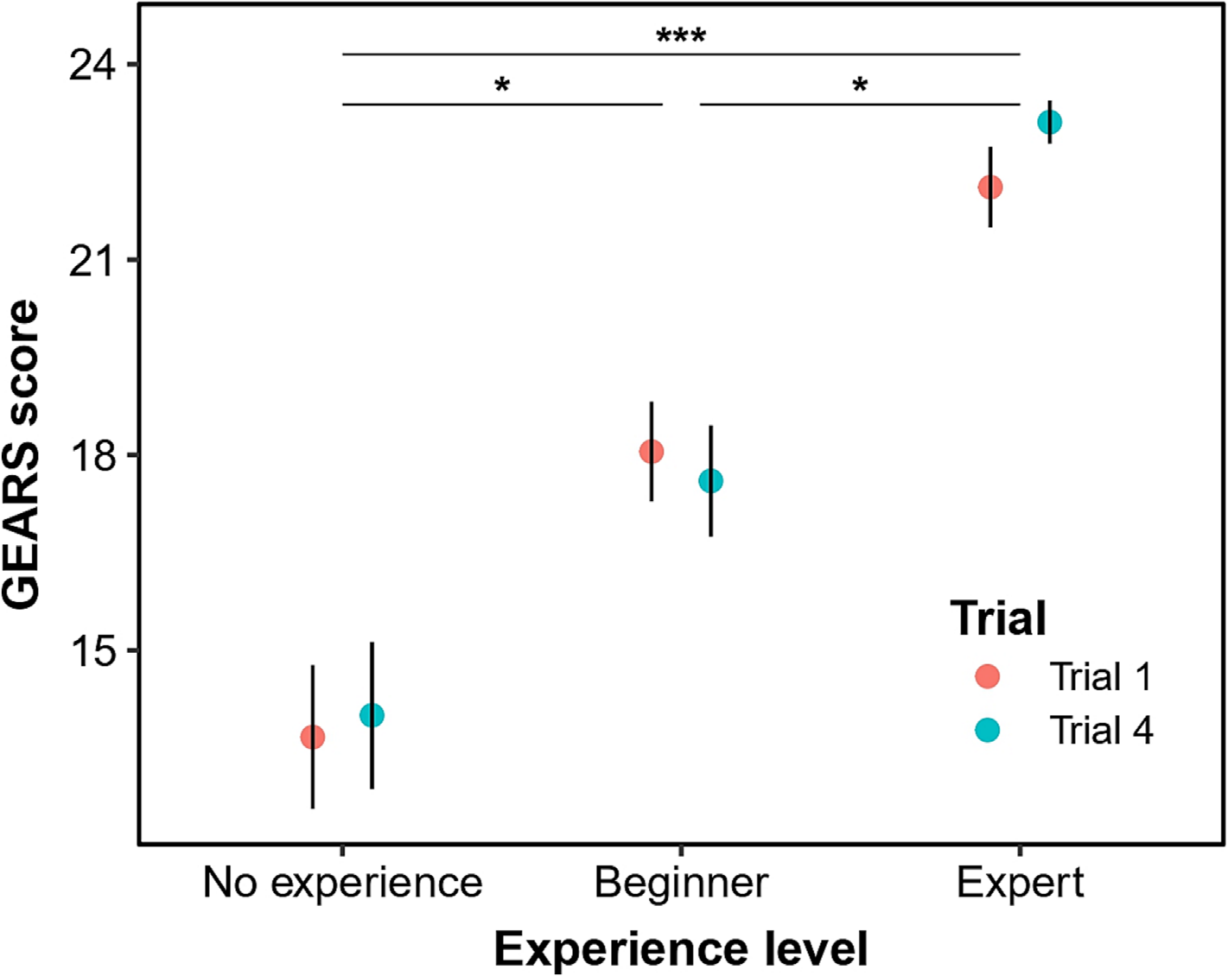
Mean ± standard error of the Global Evaluative Assessment of Robotic Surgeons (GEARS) scores per participant group comparing the first with the last tumour. Significant differences between participant groups * with p.

In order to show the potential of the model to improve robotic skills over time, we compared GEARS scores from the first tumour with the last tumour in a linear mixed model, which included the individual and the scoring expert as random factors. A tendency towards improving GEARS score could be seen in the ‘no experience’ group and especially in the expert group (Figure 4), with GEARS scores improving by 1.0 point between Trials 1 and 4. Nevertheless, there was no statistically significant improvement between trials (F = 2.00, *p* = 0.16).

## 4 DISCUSSION

The authors sought to validate a new silicone 3D kidney tumour enucleation model for training for robotic assisted partial nephrectomy and for examination of skills before performing surgery on patients autonomously. In our cohort of 36 participants, the 3D silicone kidney tumour model developed by Lazarus 3D was rated to be realistic and useful, thereby demonstrating preliminary face and content validity. NASA‐TLX scores for all participants were below the pre‐set threshold, thus rendering the simulation to be of an acceptable difficulty level. The expert group also showed a significantly shorter resection time and better surgical outcomes for all measured outcome parameters except for renal pelvis perforation. This proven association between performance and experience level makes differentiating novices from experts possible, thus confirming preliminary content validity.

Operative skill is hard to assess on a model. We believe that motion analyses are important parameters to evaluate surgical skill and efficiency in a confined space. We therefore used the validated GEARS score to evaluate surgical performance.

Expert and no experience participants performing a simulated enucleation of the four tumour models showed indication of surgical improvement between the first and last tumour, as measured with the GEARS scoring system, but this was not statistically significant. This could be explained by the fact that the fourth tumour was objectively more difficult than the first, due to its largely endophytic nature.

We could therefore not adequately demonstrate preliminary construct validity.

Participants' reactions were almost uniformly positive. Recurrent comments were about the absence of bleeding that blunt dissection was difficult because of the viscosity of the silicone tissue and that no in‐depth or 3D images of the tumours were shown before starting simulation.

Several studies exist showing face^6^, ^7^, ^8^, ^9^, ^10^, ^14^ and content validity for biological and 3D‐printed models in robotic training, though most of these did not evaluate construct validity. Most of these studies used non‐commercial, self‐developed models, which are heterogenous, expensive and hard to reproduce. Taylor et al.^8^ made in vivo and ex vivo injectable agarose mixture pseudotumours but did not validate their model. Hung et al.^7^ developed a polystyrene pseudotumour that was embedded into a porcine kidney, proving face and content validity. The model described by Monda et al. in 2018^10^ was the only kidney tumour model for which construct validity was effectively proven. Our study is the first to demonstrate face and content validity, as well as to try to demonstrate preliminary construct validity of a potentially commercially available and fully 3D‐printed kidney tumour model for robotic training.^14^

There are currently no studies comparing 3D‐printed kidney tumour models to biological models or digital simulation for robotic training.^15^, ^16^ We believe commercially available 3D‐printed tumour models to be a valid and important option in training for RAPN. Clear advantages over biological models are unlimited shelf‐life, the possibility to create specific designs, a lower cost and logistical ease. There are also advantages over digital simulation or virtual reality: lower cost, no need for a simulator, more realistic handling of tissue and more detail in the resection.

A limitation of this study was the relatively small number of participants limiting statistical power.

By design, this study was limited to tumour enucleation. The model could be validated for other parts of a RAPN as well. Some of our participants tried performing a renorraphy on our model, with good success. Other future goals should be to create a more diverse offer of tumour morphology, for example, creating models mimicking cystous tumours.

In surgical training, the balance between providing surgical training experience while, at the same time, ensuring patient safety has always been a challenge. It is well established that surgeons performing RAPN are confronted with a steep learning curve.^17^ After 100–150 cases, the reduction in warm ischemia times reaches a plateau, but complication rates keep on diminishing with increasing experience. Therefore, especially for partial nephrectomy, simulation and model training are very important.

Fixed surgical curricula including simulation and model training have been shown to improve patient outcomes during the learning curve of the surgeon and could therefore help close this gap.^18^

Given the growing popularity of dedicated robotic training centres and the possibility to self‐finance robotic simulation, the need for easily available and validated training models will only increase.

Prices of 3D‐printed models, when mass produced, may lower significantly, thereby making a broad implementation into resident or consultant robotic training in the future possible.

In conclusion, commercially available 3D‐printed models may offer an opportunity in a standardized curriculum to prepare the surgeon for the first live case.

## 5 CONCLUSIONS

We have demonstrated face and content validity of the first potentially commercially available, fully 3D‐printed kidney tumour model as a training and examination model for tumour enucleation in robot‐assisted partial nephrectomies. This model, when embedded in a standardized curriculum, could be helpful to minimize the learning curve of future kidney surgeons.

## AUTHOR CONTRIBUTIONS

Friedrich‐Carl von Rundstedt and Thomas Hermans designed the study. Thomas Hermans collected the data. Joren M. Snoeks performed all data analyses and statistics. Friedrich‐Carl von Rundstedt, Thomas Steiner, Christoph Wiesner and Frank vom Dorp gave expert advice and performed final corrections. All authors read and approved of the final manuscript.

## CONFLICT OF INTEREST STATEMENT

The authors declare no conflicts of interest.

## APPENDIX A

**TABLE A1:** Outcome parameters from this study, what they were used for and how they were measured.

Assessment	Outcome parameter	Metric
Operative demand	Mental demand	0–100 score from participant
	Physical demand	0–100 score from participant
	Temporal demand	0–100 score from participant
	Performance	0–100 score from participant
	Effort	0–100 score from participant
	Frustration	0–100 score from participant
	Overall	0–100 score from participant
Content validity	Training usefulness	0–10 score from participant
	RAPN training usefulness	0–10 score from participant
	Exam usefulness	0–10 score from participant
Face validity	Comparability	0–10 score from participant
Construct validity	GEARS A	Expert‐assessed score 0–50
	GEARS B	Expert‐assessed score 0–50
Content validity	Depth of tumour incision	Millimetres
	Excised parenchyma	Grammes
	Resection time	Minutes
	Residual tumour	Presence/absence
	Tumour spillage	Presence/absence
	Injury	Presence/absence
	Pelvis perforation	Presence/absence

Abbreviation: GEARS, Global Evaluative Assessment of Robotic Surgeons.

**TABLE A2:** Results of the statistical models evaluating differences between participant groups. Mixed models contain trial and individual as random factors. The GEARS mixed model contained scoring expert as an additional random factor. GLMM = generalized linear mixed model. η^2^ = eta‐squared coefficient. KW χ^2^ = Kruskal–Wallis chi‐squared test statistic. LRT χ^2^ = likelihood ratio test chi‐squared test statistic. r^2^_m_ and r^2^_c_ represent, respectively, marginal and conditional pseudo‐R‐squared coefficients.

Response variable	Effect size (%)	Test statistic	*p*‐Value
Kruskal–Wallis	η^2^	KW χ^2^	
Training usefulness	18.90	8.25	0.02
RAPN training usefulness	1.93	2.64	0.27
Exam usefulness	3.66	3.21	0.20
Linear model	r^2^	F	
Comparability	3.21	0.55	0.58
Mental demand	13.35	2.54	0.09
Log (physical demand)	36.37	9.43	<0.001
Temporal demand	6.39	1.13	0.34
Performance	18.78	3.81	0.03
Effort	8.08	1.45	0.25
Log (frustration)	25.11	5.53	<0.01
Overall	17.94	3.61	0.04
Linear mixed model	r^2^_m_/r^2^_c_	F	
GEARS	41.91/83.99	14.14	<0.001
Depth of tumour incision	7.85/39.22	4.72	0.02
GLMM gamma distribution	r^2^_m_/r^2^_c_	LRT χ^2^	
Excised parenchyma	1.95/59.60	221.80	<0.001
Resection time	17.69/64.54	74.89	<0.001
GLMM binomial distribution	r^2^_m_/r^2^_c_	LRT χ^2^	
Residual tumour tissue	90.86/91.54	16.65	<0.001
Tumour spillage	14.31/47.86	12.68	<0.01
Tumour capsula injury	14.88/64.96	11.89	<0.01
Renal pelvis perforation	2.26/42.05	1.92	0.38

Abbreviations: GEARS, Global Evaluative Assessment of Robotic Surgeons; GLMM, generalized linear mixed model; RAPN, robot‐assisted partial nephrectomy.

**TABLE A3:** Mean ± standard error of the difficulty of each 3D‐printed robotic‐assisted partial nephrectomy training trial, as scored by each participant on a scale to 10, by participant experience level.

Trial	No experience	Beginner	Expert	Overall
Trial 1	4.6 ± 0.5	4.8 ± 0.7	2.7 ± 0.4	4.1 ± 0.4
Trial 2	4.2 ± 0.4	3.5 ± 0.5	2.7 ± 0.4	3.5 ± 0.3
Trial 3	3.8 ± 0.9	3.3 ± 1.0	3.6 ± 0.7	3.6 ± 0.3
Trial 4	8.7 ± 0.4	8.2 ± 0.6	6.5 ± 0.7	7.9 ± 0.3

**TABLE A4:** Results of the statistical models evaluating differences between different types of usefulness scores, Nasa Task Load Index (NASA‐TLX) scores, trial difficulties, respectively. Additionally, it shows the results of the statistical models evaluating differences between the trial difficulty scores of different participant groups. η^2^ = eta‐squared coefficient. KW χ^2^ = Kruskal–Wallis chi‐squared test statistic.

Response variable	Predictor variable	Effect size (%)	Test statistic	P‐value
Kruskal–Wallis		η^2^	KW χ^2^	
Usefulness score	Usefulness type	0.09	2.09	0.35
Linear model		r^2^	F	
Difficulty score	Trial	48.29	42.64	<0.001
Trial 1 difficulty	Experience level	17.49	3.50	0.04
Trial 2 difficulty	Experience level	15.68	2.98	0.07
Trial 3 difficulty	Experience level	1.74	0.28	0.76
Trial 4 difficulty	Experience level	19.93	3.98	0.03
